# Spending Operators and Weak Power-Port Decompositions for Path-Dependent Entropic Lagrangians

**DOI:** 10.3390/e28101139

**Published:** 2026-10-16

**Authors:** Huilong Ren

**Affiliations:** College of Civil Engineering, Tongji University, Shanghai 200092, China; hlren@tongji.edu.cn

**Keywords:** terminal variation, cocycle calibration, weak Leibniz rule, chemical-potential weighting, local species balance, Cahn–Hilliard equation, 49J40, 49S05, 35K55, 35D30, 46N20, 74A15

## Abstract

Path-dependent entropic Lagrangians formulate irreversible evolution through terminal variations of accumulated process power. This paper establishes a mathematical basis for calibrated terminal spending and weak diffusion power-port decompositions. Endpoint calibration and cocycle additivity determine a unique oriented spending increment, while local selector fields resolve its channel contributions. The diffusion power-port decomposition extends from Sobolev fields with normal-trace boundary power to finite-energy fields through a distributional balance component. These constructions are combined in a synchronized mixed thermo-diffusion functional. A Cahn–Hilliard specialization illustrates the weak channel structure and the associated energy balance.

## 1. Introduction

In mechanics, variational principles connect stored energy and virtual work with governing equations and natural boundary conditions within a unified structure. Extending this structure to irreversible evolution requires dissipation, transport, and history to be incorporated while preserving the distinction between reversible storage and process power along the physical trajectory. Onsager’s reciprocal relations and Biot’s irreversible thermodynamics express dissipative evolution through force–rate pairings, while generalized standard materials and incremental constitutive principles encode the material response by convex functions of rates or internal variables [1,2,3,4,5,6]. Generalized-gradient systems extend this setting to nonlinear and nonsmooth evolutions and endow them with a precise energy–dissipation structure once the state space, energy, and balance operator have been fixed [7,8]. These formulations systematically characterize constitutive evolution and stability; accumulated process power, however, is normally evaluated along the resulting trajectory rather than treated as the object of an independently calibrated terminal variation.

A complementary line of development formulates irreversible systems in terms of power exchange and nonconservative action principles. Port-Hamiltonian and Stokes–Dirac structures organize bulk–boundary power exchange and have recently been developed in Hilbert-space settings that make the boundary dualities explicit [9,10,11]. Doubled-variable actions and constrained thermodynamic principles enlarge the variation space in order to accommodate nonconservative forces and entropy production [12,13,14,15]. Recent extensions treat thermodynamic fluxes as independent variables, incorporate objective heat-flux rates, and formulate general dissipative fluid systems by differential forms [16,17]. Taken together, these approaches establish broad variational settings for irreversible mechanisms. The specific question addressed here is the first-order allocation, among distinct terminal channels, of process power accumulated along the physical trajectory.

Diffusion introduces an additional analytical difficulty because the local power decomposition must remain meaningful at the weak regularity natural to the evolution problem. The Cahn–Hilliard equation can be derived from microforce balances, energetic variational principles, and mixed rate formulations [18,19,20,21,22]. In weak solutions, however, the natural regularity is often only a chemical potential in $H^{1}\left(\Omega \right)$ and a flux in $L^{2}\left(\Omega ;R^{d}\right)$. The classical product formula for a potential-weighted flux must then be interpreted distributionally, and the normal trace must be retained when boundary power is present. The corresponding tools come from H(div) theory, divergence-measure fields, and modern pairing formulas [23,24,25,26]. Accordingly, the weak product structure determines whether the balance-side and entropy-side contributions of the diffusion port are well defined in the function spaces in which the evolution is formulated.

The Path-Dependent Entropic Lagrangian framework (PDEL) was introduced for irreversible thermomechanical systems and subsequently developed as a path-dependent variational formulation of irreversible thermodynamics [27,28]. A central operation in PDEL is the channelwise routing of accumulated process power, while balance laws and thermodynamic conjugacies are generated within the same variational structure. The present paper isolates the mathematical structures needed for that operation. First, synchronization of terminal variables determines only a diagonal set and does not fix the amplitude of the channel differential. Second, terminal selectors must be local test fields if their stationarity is to yield local equations. Third, the diffusion port requires a weak Leibniz decomposition compatible with finite-energy fields. Finally, the chemical-potential-weighted species channel must be connected to local conservation through the constitutive structure of zero-potential states, with an independent weak formulation available when the regularity needed for that argument is absent.

Two neighboring variational frameworks help position the present construction. In the standard Onsager principle, the stationarity of a Rayleighian determines rates or fluxes subject to prescribed kinematics and conservation constraints [1,2,6]; it is therefore compatible with the potential-based constitutive closures considered here. The PDEL terminal operation acts at a different level: it starts from the accumulated power history generated by the physical trajectory and resolves its calibrated endpoint differential into independent terminal channels. Port-Hamiltonian and Stokes–Dirac formulations share the bulk–boundary effort–flux pairing and adjoint structure adopted below, including their weak realizations [9,10,11]. The construction developed here links these power pairings to calibrated terminal histories and their channelwise differentials.

The analysis establishes a mathematical basis for these terminal operations and for the associated diffusion power-port decomposition. Cocycle additivity and endpoint calibration determine the oriented spending increment; local selector fields resolve the corresponding channel contributions. For diffusion, the decomposition is extended from H(div) fields with normal-trace boundary power to finite-energy fields through a distributional balance component. The weighted species channel is then related to the primitive conservation law through explicit recovery conditions, while an auxiliary mixed weak formulation covers the natural dual setting. The same ingredients are assembled in a synchronized thermo-diffusion functional and illustrated by a Cahn–Hilliard realization. The arguments draw on standard analytical tools—the fundamental theorem of calculus, annihilator identities, Sobolev product formulas, and Fenchel duality—to formulate the PDEL terminal and power-port operations precisely.

The remainder of the paper is outlined as follows. Section 2 fixes the analytical setting and distinguishes vertical path variation from the history-locked terminal derivative. Section 3 establishes the calibrated spending rule and its localized selector fields. Section 4 develops the Sobolev and finite-energy diffusion-port decompositions. Section 5 gives the constitutive recovery of local balance and the auxiliary mixed weak formulation. Section 6 records the energy balance and its relation to process power. Section 7 constructs the synchronized mixed thermo-diffusion functional, and Section 8 gives the Cahn–Hilliard realization. Section 9 summarizes the conclusions; auxiliary multichannel and measure-valued extensions are collected in the appendices.

## 2. Analytical Setting

### 2.1. Power and Constitutive Contact

Let *X* be a real reflexive Banach space with dual $X^{*}$. A path

(1) $$z\in W^{1,1}\left(0,T;X\right),\;v\left(t\right):=\dot{z}\left(t\right)\in X\;\mathrm{for}\;\mathrm{almost}\;\mathrm{everywhere}\;t,$$

has a conjugate effort $r\left(t\right)\in X^{*}$. The scalar power is

(2) $$D\left(t\right):={\langle r(t),v(t)\rangle }_{X^{*},X}.$$

Its sign is constitutive.

When constitutive contact is represented by a proper, convex, lower-semicontinuous rate-power function R:X→[0,+∞] with R(0)=0, the standard Fenchel relation gives

(3) $$r\in \partial R\left(v\right)\;⟺\;\langle r,v\rangle =R\left(v\right)+R^{*}\left(r\right)\geq 0.$$

Constitutive contact identifies the nonnegative process power [7,29,30]; terminal orientation and spatial decomposition are treated separately below. In the continuum examples below, the local constitutive functions are called flux power densities.

### 2.2. Vertical and Terminal Variations

A vertical path variation in an admissible path class X is generated by a curve

(4) $$z_{\varepsilon }=z+\varepsilon \eta ,\;\eta \in T_{z}X.$$

For a smooth integrand P=P(z,v,t), differentiation of a history with a moving endpoint gives

(5) $$\begin{array}{cc} \; & {\left.\frac{\;d}{\;d\varepsilon }\right|}_{\varepsilon =0}\int_{t_{0}}^{t+\varepsilon \xi }P\left(z_{\varepsilon }\left(\tau \right),{\dot{z}}_{\varepsilon }\left(\tau \right),\tau \right)\;d\tau \\ \; & \;=\int_{t_{0}}^{t}\left[D_{z}P\left(z,\dot{z},\tau \right)\left[\eta \right]+D_{v}P\left(z,\dot{z},\tau \right)\left[\dot{\eta }\right]\right]\;d\tau +P\left(z\left(t\right),\dot{z}\left(t\right),t\right)\xi , \end{array}$$

whenever differentiation under the integral is justified. The terminal operation used in this paper is the restriction η=0 in (5).

For $D\in L^{1}\left(t_{0},T\right)$, set

(6) $$H_{D}\left(t\right):=\int_{t_{0}}^{t}D\left(\tau \right)\;d\tau .$$

Then, $H_{D}\in W^{1,1}\left(t_{0},T\right)$, and ${\dot{H}}_{D}=D$ almost everywhere. If the power is generated along a realized path, write $D_{z}\left(t\right)=\langle r_{z}\left(t\right),\dot{z}\left(t\right)\rangle$ and $H\left[z_{\leq t}\right]=H_{D_{z}}\left(t\right)$.

**Definition 1** (History-locked terminal derivative)**.** *Fix $t\in (t_{0},T)$, a realized history, and its associated power $D_{z}\in L^{1}\left(t_{0},T\right)$. The history-locked terminal derivative is*

(7) $$\partial_{t}^{\mathrm{hl}}H\left[z_{\leq t}\right]:=\lim_{h\to 0,\;t+h\in (t_{0},T)}\frac{H_{D_{z}}\left(t+h\right)-H_{D_{z}}\left(t\right)}{h},$$

*whenever the limit exists. The function $D_{z}$ is held fixed in this operation; no vertical perturbation of z or of the constitutive law is taken inside the accumulated history.*

At every Lebesgue point of $D_{z}$,

(8) $$\partial_{t}^{\mathrm{hl}}H\left[z_{\leq t}\right]=D_{z}\left(t\right).$$

The distinction between vertical and terminal directions parallels the separation of path and time derivatives in nonanticipative path calculus [31].

## 3. Calibrated Terminal Spending

### 3.1. Oriented History Extension

Let

(9) $$\Delta :=\left\{\left(t,t\right):t\in \left(t_{0},T\right)\right\}.$$

If a $C^{1}$ function $\tilde{\Gamma }$ vanishes on Δ, then $D\tilde{\Gamma }\left(t,t\right)=a\left(t\right)\left(1,-1\right)$ for some scalar a(t), because the differential annihilates the tangent direction (1,1). The diagonal condition determines the conormal direction but not its amplitude. Endpoint calibration is therefore an additional datum.

**Definition 2**  (Calibrated oriented history extension). *Let $D\in L^{1}\left(t_{0},T\right)$. A map $\Gamma :{\left[t_{0},T\right]}^{2}\to R$ is a calibrated oriented history extension of D if*

(i) *Γ(a,c)=Γ(a,b)+Γ(b,c) for all a,b,c;*
(ii) *For every fixed b, the map a↦Γ(a,b) is absolutely continuous;*
(iii) *At every Lebesgue point t of D,*
  (10) $$\lim_{h\to 0}\frac{\Gamma (t+h,t)}{h}=D\left(t\right),$$
  *whenever $t+h\in [t_{0},T]$.*

**Theorem 1** (Uniqueness and spending rule)**.** *For every $D\in L^{1}\left(t_{0},T\right)$, there is exactly one calibrated oriented history extension:*

(11) $$\Gamma_{D}\left(t_{s},t_{c}\right)=H_{D}\left(t_{s}\right)-H_{D}\left(t_{c}\right)=\int_{t_{c}}^{t_{s}}D\left(\tau \right)\;d\tau .$$

*At every Lebesgue point t of D,*

(12) $$D\Gamma_{D}\left(t,t\right)\left[\delta t_{s},\delta t_{c}\right]=D\left(t\right)\delta t_{s}-D\left(t\right)\delta t_{c}.$$

*The common direction is null: $D\Gamma_{D}\left(t,t\right)\left[\xi ,\xi \right]=0$.*

**Proof.**  Let $F\left(t\right)=\Gamma \left(t,t_{0}\right)$. The cocycle identity gives Γ(a,b)=F(a)−F(b) and also $F(t_{0})=0$. By absolute continuity and (10), $F^{\prime }=D$ almost everywhere; hence, $F=H_{D}$. Differentiating (11) at (t,t) gives (12). □

The cocycle identity fixes the difference structure, while endpoint calibration determines its amplitude and orientation.

The directly usable notation is

(13) $$\delta^{\mathrm{ch}}\int_{t_{0}}^{t}D\left(\tau \right)\;d\tau =D\left(t\right)\delta t_{s}-D\left(t\right)\delta t_{c}.$$

Here, $\delta^{\mathrm{ch}}$ denotes the diagonal differential of the calibrated extension. Equivalently, define

(14) $$H_{\varepsilon }:=H_{D}\left(t\right)+\Gamma_{D}\left(t+\varepsilon \delta t_{s},t+\varepsilon \delta t_{c}\right).$$

Then, $H_{0}=H_{D}\left(t\right)$, and $H_{\varepsilon }^{\prime }{|}_{\varepsilon =0}$ is the right-hand side of (13). The calibrated two-channel spending rule extends naturally to multiple terminal channels through zero-sum coupling matrices, as shown in Appendix A.

### 3.2. Spending Operator and Trajectory-Generated Directions

For a fixed rate v∈X, set

(15) $$K_{v}:=\left\{(v\xi ,\xi ):\xi \in R\right\}\subset X\times R,$$

and define

(16) $$S_{v}:X^{*}\to X^{*}\times R,\;S_{v}\left(r\right):=\left(r,-\langle r,v\rangle \right).$$

A direct calculation gives

(17) $$K_{v}^{⊥}=\mathrm{Ran}S_{v}=\left\{\left(r,-\langle r,v\rangle \right):r\in X^{*}\right\}.$$

Consequently, if $D=\langle r,v\rangle$ and $\eta =v\delta t_{c}$, then

(18) $$D\delta t_{s}-D\delta t_{c}=D\delta t_{s}-\langle r,\eta \rangle .$$

The relation $\eta =v\delta t_{c}$ selects one direction generated by the realized trajectory. In vector mechanics, arbitrary virtual displacements remain independent directions. Moreover, the scalar *D* does not determine *r*: every functional annihilating *v* can be added to *r* without changing *D*.

### 3.3. Localized Selector Fields

Let I⊂R be an open interval, let Ω have finite measure, and let $p\in C(I;L^{1}\left(\Omega \right))$ be a jointly measurable power density. Fix $t_{0}\in I$. For measurable endpoint fields $t_{s},t_{c}:\Omega \to I$ with ranges in a common compact subinterval of *I* containing $t_{0}$, define

(19) $$\Gamma_{p}\left[t_{s},t_{c}\right]:=\int_{\Omega }\left[\int_{t_{0}}^{t_{s}\left(x\right)}p\left(x,\tau \right)\;d\tau -\int_{t_{0}}^{t_{c}\left(x\right)}p\left(x,\tau \right)\;d\tau \right]\;dx.$$

**Theorem 2** (Localized spending rule)**.** *Fix t∈I, and let $\delta t_{s},\delta t_{c}\in L^{\infty }\left(\Omega \right)$. For sufficiently small ε, set*

(20) $$t_{s}^{\varepsilon }=t+\varepsilon \delta t_{s},\;t_{c}^{\varepsilon }=t+\varepsilon \delta t_{c}.$$

*Then,*

(21) $${\left.\frac{\;d}{\;d\varepsilon }\right|}_{\varepsilon =0}\Gamma_{p}\left[t_{s}^{\varepsilon },t_{c}^{\varepsilon }\right]=\int_{\Omega }p\left(x,t\right)\left(\delta t_{s}-\delta t_{c}\right)\;dx.$$

**Proof.**  It suffices to treat one endpoint. For $h\in L^{\infty }\left(\Omega \right)$, let

$$F_{\varepsilon }\left(h\right):=\int_{\Omega }\int_{t}^{t+\varepsilon h(x)}p\left(x,\tau \right)\;d\tau \;dx,\;M:={∥h∥}_{L^{\infty }}.$$

Fubini’s theorem yields

$$\begin{array}{cc} \left|\frac{F_{\varepsilon }\left(h\right)}{\varepsilon }-\int_{\Omega }p\left(x,t\right)h\left(x\right)\;dx\right|\; & \leq \frac{1}{\left|\varepsilon \right|}\int_{t-|\varepsilon |M}^{t+|\varepsilon |M}{∥p(\cdot ,\tau )-p(\cdot ,t)∥}_{L^{1}}\;d\tau \\ \; & \leq 2M\underset{|\tau -t|\leq |\varepsilon |M}{\sup}{∥p(\cdot ,\tau )-p(\cdot ,t)∥}_{L^{1}}, \end{array}$$

which tends to zero. Applying the calculation to both endpoints proves the claim. □

The estimate shows why continuity with values in $L^{1}\left(\Omega \right)$ is sufficient: only the behavior of the power density in a shrinking time neighborhood of the terminal point is used. No pointwise continuity in *x* is required.

For interior localization at a fixed time, the selectors are taken independently in

(22) $$\delta t_{c},\delta t_{s}\in C_{c}^{\infty }\left(\Omega \right).$$

They are virtual test fields with the dimension of time. If $\int_{\Omega }\left(A_{c}\delta t_{c}+A_{s}\delta t_{s}\right)\;dx=0$ for all such pairs, then $A_{c}=A_{s}=0$ in $D^{\prime }\left(\Omega \right)$. The weak balance below is tested directly against the full space $H^{1}\left(\Omega \right)$.

## 4. Diffusion Power-Port Decomposition

Let $\Omega \subset R^{d}$ be a bounded Lipschitz domain. The chemical potential is denoted by μ and the diffusion flux by j. The smooth identity

(23) div(μj)=μdivj+j·∇μ

is a local Leibniz rule. Its interpretation as a channel decomposition requires a regularity class, a boundary convention, and independent terminal selectors.

### 4.1. Sobolev Fields and Boundary Power

Recall

(24) $$H\left(\mathrm{div};\Omega \right):=\left\{j\in L^{2}\left(\Omega ;R^{d}\right):\mathrm{div}j\in L^{2}\left(\Omega \right)\right\}.$$

For j∈H(div;Ω), its normal trace $\gamma_{n}j\in H^{-1/2}\left(\partial \Omega \right)$ satisfies

(25) $$\int_{\Omega }j\cdot \nabla \eta \;dx+\int_{\Omega }\left(\mathrm{div}j\right)\eta \;dx={\langle \gamma_{n}j,\gamma \eta \rangle }_{H^{-1/2},H^{1/2}}$$

for every $\eta \in H^{1}\left(\Omega \right)$ [23,32].

**Theorem 3** (Sobolev product rule and boundary port)**.** *Let $\mu \in H^{1}\left(\Omega \right)$ and j∈H(div;Ω). Then μj, μdivj, and j·∇μ belong to $L^{1}\left(\Omega \right)$, and*

(26) $$\mathrm{div}\left(\mu j\right)=\mu \mathrm{div}j+j\cdot \nabla \mu \;\mathrm{in}\;D^{\prime }\left(\Omega \right).$$

*Moreover,*

(27) $$\int_{\Omega }\mu \mathrm{div}j\;dx+\int_{\Omega }j\cdot \nabla \mu \;dx={\langle \gamma_{n}j,\gamma \mu \rangle }_{H^{-1/2},H^{1/2}}.$$

**Proof.**  The integrability statements follow from Hölder’s inequality. For $\varphi \in C_{c}^{\infty }\left(\Omega \right)$, the function μφ belongs to $H_{0}^{1}\left(\Omega \right)$. Hence,

$$0=\int_{\Omega }\left(\mathrm{div}j\right)\mu \varphi \;dx+\int_{\Omega }j\cdot \nabla \left(\mu \varphi \right)\;dx,$$

and expansion of the gradient gives (26). Equation (27) is (25) with η=μ. □

Set

(28) $$P_{c}:=\mu \mathrm{div}j,\;P_{s}:=j\cdot \nabla \mu .$$

The subscripts refer to the conserved-variable and entropy channels. Equation (27) shows that their spatial integrals sum to boundary chemical power. They cancel globally only for a closed port, for example, $\gamma_{n}j=0$.

### 4.2. Channelwise Terminal Variation

Assume that $P_{c},P_{s}\in C\left(I;L^{1}\left(\Omega \right)\right)$ on an open time interval *I*. For measurable endpoint fields $t_{c},t_{s}:\Omega \to I$ with ranges in a common compact subinterval of *I* containing $t_{0}$, define

(29) $$\begin{array}{cc} \Gamma_{P}\left[t_{c},t_{s}\right]:= & \int_{\Omega }\int_{t_{0}}^{t_{c}\left(x\right)}P_{c}\left(x,\tau \right)\;d\tau \;dx\\ \; & +\int_{\Omega }\int_{t_{0}}^{t_{s}\left(x\right)}P_{s}\left(x,\tau \right)\;d\tau \;dx. \end{array}$$

On the synchronization diagonal,

(30) $$\Gamma_{P}\left[t,t\right]=\int_{\Omega }\int_{t_{0}}^{t}\mathrm{div}\left(\mu j\right)\left(x,\tau \right)\;d\tau \;dx.$$

**Theorem 4** (Diffusion-port terminal rule)**.** *Fix t∈I, let $\delta t_{c},\delta t_{s}\in L^{\infty }\left(\Omega \right)$, and set*

(31) $$G_{\varepsilon }:=\Gamma_{P}\left[t+\varepsilon \delta t_{c},t+\varepsilon \delta t_{s}\right].$$

*Then,*

(32) $${\left.\frac{\;dG_{\varepsilon }}{\;d\varepsilon }\right|}_{\varepsilon =0}=\int_{\Omega }\left(\mu \mathrm{div}j\;\delta t_{c}+j\cdot \nabla \mu \;\delta t_{s}\right)\left(x,t\right)\;dx.$$

**Proof.**  Apply Theorem 2 separately to the two component histories in (29). □

At ε=0, the two endpoint fields coincide, and (30) recovers the original accumulated diffusion port. The selectors distinguish only the first-order channel directions. Both channels arise from the single local identity (23).

The local notation used in applications is therefore

(33) $$\delta^{\mathrm{ch}}\int_{t_{0}}^{t}\mathrm{div}\left(\mu j\right)\;d\tau =\mu \mathrm{div}j\;\delta t_{c}+j\cdot \nabla \mu \;\delta t_{s}.$$

Equation (33) is the local shorthand for the tested identity (32).

### 4.3. Finite-Energy Fields

For weak diffusion fields, one often has only

(34) $$\mu \in H^{1}\left(\Omega \right),\;j\in L^{2}\left(\Omega ;R^{d}\right).$$

Then, $\mu j\in L^{1}$, and $j\cdot \nabla \mu \in L^{1}$, but the ordinary product μdivj need not be defined.

**Definition 3** (Finite-energy balance component)**.** *For *(34)*, define*

(35) $$\mu ⋄\mathrm{div}j:=\mathrm{div}\left(\mu j\right)-j\cdot \nabla \mu \;\mathrm{in}\;D^{\prime }\left(\Omega \right).$$

*Equivalently, for $\varphi \in C_{c}^{\infty }\left(\Omega \right)$,*

(36) $$\begin{array}{cc} \langle \mu ⋄\mathrm{div}j,\varphi \rangle := & -\int_{\Omega }\mu j\cdot \nabla \varphi \;dx\\ \; & -\int_{\Omega }\varphi \;j\cdot \nabla \mu \;dx. \end{array}$$

By construction,

(37) $$\mathrm{div}\left(\mu j\right)=\mu ⋄\mathrm{div}j+j\cdot \nabla \mu \;\mathrm{in}\;D^{\prime }\left(\Omega \right).$$

If j∈H(div;Ω), then μ⋄divj=μdivj in distributions; so, (37) extends (26). A complementary measure-valued decomposition for BV potentials and divergence-measure fluxes, including interfacial power contributions, is provided in Appendix B.

For local selectors $\delta t_{c},\delta t_{s}\in C_{c}^{\infty }\left(\Omega \right)$, the finite-energy channel differential is

(38) $$\langle \mu ⋄\mathrm{div}j,\delta t_{c}\rangle +\int_{\Omega }j\cdot \nabla \mu \;\delta t_{s}\;dx.$$

This is the rigorous replacement of the right-hand side of (33) at finite energy.

The test-space continuity follows directly from

(39) $$|\langle \mu ⋄\mathrm{div}j,\varphi \rangle |\leq {∥j∥}_{L^{2}}\left({∥\mu ∥}_{L^{2}}{∥\nabla \varphi ∥}_{L^{\infty }}+{∥\nabla \mu ∥}_{L^{2}}{∥\varphi ∥}_{L^{\infty }}\right).$$

If $\mu \in L^{2}\left(I;H^{1}\right)$ and $j\in L^{2}\left(I;L^{2}\right)$, each fixed-test pairing is integrable in time; so, its history primitive has a terminal derivative at almost every time. The history derivative is taken after spatial testing. The density-level scalar histories in Section 7 use the stronger regularity specified there.

**Proposition 1** (Strong–weak stability)**.** *Suppose*

(40) $$\mu_{n}\to \mu \;\mathrm{strongly}\;\mathrm{in}\;H^{1}\left(\Omega \right),\;j_{n}⇀j\;\mathrm{weakly}\;\mathrm{in}\;L^{2}\left(\Omega ;R^{d}\right).$$

*Then,*

(41) $$\mu_{n}⋄\mathrm{div}j_{n}⟶\mu ⋄\mathrm{div}j\;\mathrm{in}\;D^{\prime }\left(\Omega \right),$$

*and $j_{n}\cdot \nabla \mu_{n}\to j\cdot \nabla \mu$ in distributions.*

**Proof.**  For a fixed smooth compactly supported test function, both terms in (36) are products of one strongly convergent $L^{2}$ factor and one weakly convergent $L^{2}$ factor. The same argument applies to the force–flux product. □

At H(div) regularity, spatially constant selectors and a closed normal port give, by (27),

(42) $$\delta^{\mathrm{ch}}\int_{\Omega }\int_{t_{0}}^{t}\mathrm{div}\left(\mu j\right)\;d\tau \;dx=D_{d}\left(t\right)\left(\delta t_{c}-\delta t_{s}\right),\;D_{d}\left(t\right):=-\int_{\Omega }j\cdot \nabla \mu \;dx.$$

A constitutive law determines the sign of $D_{d}$. For example, if

(43) $$j=-M\nabla \mu ,\;M=M^{T}⪰0,$$

then the local diffusion power density is

(44) −j·∇μ=∇μ·M∇μ≥0.

## 5. Recovery of Local Species Balance

Let

(45) $$R^{d}:=\dot{c}+\mathrm{div}j$$

denote the species residual for a regular diffusion field. The conserved-variable selector in (33), together with the terminal chemical power $\mu \dot{c}\;\delta t_{c}$, gives

(46) $$\mu R^{d}=0.$$

This coefficient identifies the primitive residual $R^{d}$, which is tested and assembled directly, without numerical division by μ. The following theorem gives sufficient conditions for equivalence of the weighted and unweighted equations by examining regions where the chemical potential vanishes persistently.

**Theorem 5** (Constitutive recovery of local balance)**.** *Let $Q:=\Omega \times (t_{0},T)$, let $c,\mu \in C^{1}\left(Q\right)$ and $j\in C^{1}\left(Q;R^{d}\right)$, and let ζ denote prescribed constitutive data. Define $R^{d}$ by *(45)*. Suppose that*

(i) *$\mu R^{d}=0$ in Q;*
(ii) *The flux law has the form*
  (47) j=J(x,t,c,ζ,−∇μ),J(x,t,c,ζ,0)=0;
(iii) *For every open cylinder $U\times I⋐{\mathrm{int}}_{Q}\left\{\mu =0\right\}$, there are Banach spaces $X_{U}$ and $Z_{U}$, time-independent constitutive data $\zeta_{U}$ satisfying ${\zeta |}_{U\times I}=\zeta_{U}$, and a map*
  (48) $$M_{U}\left(\;\cdot \;;\zeta_{U}\right)\in C^{1}\left(X_{U};Z_{U}\right)$$
  *such that ${c|}_{U}\in C^{1}\left(I;X_{U}\right)$, $\dot{c}{\left(t\right)|}_{U}\in T_{c(t)}A_{U}$, ${\mu |}_{U}=M_{U}\left(c{|}_{U};\zeta_{U}\right)$ in $Z_{U}$, and*
  (49) $$\mathrm{Ker}D_{c}M_{U}\left(c\left(t\right){|}_{U};\zeta_{U}\right)\cap T_{c(t)}A_{U}=\left\{0\right\}\;\mathrm{for}\;\mathrm{every}\;t\in I,$$
  *where $T_{c(t)}A_{U}$ is the admissible tangent space.*

*Then,*

(50) $$\dot{c}+\mathrm{div}j=0\;\mathrm{in}\;Q.$$

**Proof.**  At every point with μ≠0, (46) gives $R^{d}=0$. If a point belongs to the boundary of $\left\{\mu =0\right\}$, it is approached by points with μ≠0; continuity of $R^{d}$ therefore gives $R^{d}=0$ there as well. It remains to consider an open cylinder $U\times I⋐{\mathrm{int}}_{Q}\left\{\mu =0\right\}$.

On this cylinder, μ=0, and hence, ∇μ=0. Assumption (ii) gives j=0; so, divj=0 locally in distributions. The identity $M_{U}\left(c\left(t\right){|}_{U};\zeta_{U}\right)=0$ can be differentiated in $Z_{U}$ because $\zeta_{U}$ is fixed in time:

$$D_{c}M_{U}\left(c\left(t\right){|}_{U};\zeta_{U}\right)\left[\dot{c}\left(t\right){|}_{U}\right]=0.$$

The injectivity condition (49) yields $\dot{c}=0$ on U×I. Hence, $R^{d}=0$ throughout the interior of the zero set and therefore in all of *Q*. □

The injectivity condition excludes motion along a neutral family of zero-potential states. Together with zero force and zero flux, it ensures dynamical isolation on a persistent zero-potential region.

**Corollary 1** (Local energies with isolated critical states)**.** *Let $c,\mu \in C^{1}\left(Q\right)$ and $j\in C^{1}\left(Q;R^{d}\right)$. Assume *(46)*, a flux law j=J(−∇μ) with J(0)=0, and*

(51) $$\mu =f^{\prime }\left(c\right)$$

*for a function $f\in C^{1}\left(R\right)$ whose critical set $\left\{r\in R:f^{\prime }\left(r\right)=0\right\}$ is discrete. Then, *(50)* holds.*

**Proof.**  On each connected component of an open zero-potential region, continuity of *c* and discreteness of the critical set force *c* to take one constant critical value. Thus $\dot{c}=0$. Equation (47) gives j=0, and the local residual vanishes. Outside such a region the proof of Theorem 5 applies. □

For a local potential $\mu =f^{\prime }\left(c\right)$ with $f\in C^{2}$, condition (49) is satisfied at a critical state $c_{*}$ if $f^{\prime \prime }\left(c_{*}\right)\neq 0$; positivity is not required. For $f\left(c\right)=a{\left(c-c_{*}\right)}^{2}/2$, a>0, the derivative is *a*. For $f\left(c\right)={\left(c^{2}-1\right)}^{2}/4$, the critical states −1,0,1 have derivatives 2,−1,2, respectively. Corollary 1 also applies when a critical state is isolated but its second derivative vanishes.

For a gradient energy with $f\in C^{2}$ and κ>0,

(52) $$\mu =f^{\prime }\left(c\right)-\kappa \Delta c,$$

and condition (49) is the absence of an admissible kernel of the linearized operator $f^{\prime \prime }\left(c\right)-\kappa \Delta$ at a zero-potential state. A sufficient test, for homogeneous tangent boundary conditions that remove the boundary term, is

(53) $$\kappa \int_{U}{\left|\nabla h\right|}^{2}\;dx+\int_{U}f^{\prime \prime }\left(c\right){\left|h\right|}^{2}\;dx\geq \alpha {∥h∥}_{H^{1}\left(U\right)}^{2},\;\alpha >0,$$

for all admissible *h*. For example, $f^{\prime \prime }\left(c\right)\geq a_{0}>0$ suffices with homogeneous Dirichlet or Neumann tangent conditions. The test must use the actual local domain and tangent space in Theorem 5; global boundary or mass constraints do not automatically hold on every interior zero-potential region. Translation-invariant interfaces can carry neutral modes, in which case the weighted channel alone does not determine motion along the equilibrium family; actual Cahn–Hilliard front motion is governed by the balance-constrained transport dynamics [33]. Time-dependent external fields require the corresponding term $D_{\zeta }M_{U}\left[\dot{\zeta }\right]$ in the differentiated equilibrium relation and are therefore outside the stationary-parameter hypothesis of Theorem 5.

### Weak Balance and Auxiliary Mixed Formulation

The preceding recovery theorem concerns regular fields and uses the constitutive structure of persistent zero-potential states. At finite-energy regularity, local conservation is formulated directly in a dual space. Use the Gelfand triple

(54) $$V=H^{1}\left(\Omega \right)↪H=L^{2}\left(\Omega \right)≅H^{*}↪V^{*},\;Y:=L^{2}\left(\Omega ;R^{d}\right).$$

Let G:V→Y be Gη=∇η, and define the closed-port weak divergence

(55) $$B:=-G^{*}:Y\to V^{*},\;{\langle Bj,\eta \rangle }_{V^{*},V}:=-\int_{\Omega }j\cdot \nabla \eta \;dx.$$

Then,

(56) $${\langle Bj,\mu \rangle }_{V^{*},V}+{\left(j,G\mu \right)}_{Y}=0.$$

For $v\in V^{*}$, define

(57) $$R:=v+Bj\in V^{*}.$$

The primitive balance is R=0 tested against all of *V*; for regular fields and $v=\dot{c}$, its interior residual is $R^{d}$. In the mixed formulation below, λ and its variation lie in *V*, rate variations lie in $V^{*}$, and flux variations lie in *Y*. A channel selector *a* instead must multiply *V* into itself. A sufficient choice is $a\in W^{1,\infty }\left(\Omega \right)$, since

(58) $${∥a\mu ∥}_{H^{1}}\leq C\left({∥a∥}_{L^{\infty }}+{∥\nabla a∥}_{L^{\infty }}\right){∥\mu ∥}_{H^{1}}.$$

Thus, 〈R,μa〉 is defined; an arbitrary $H^{1}$ selector need not suffice. Compactly supported smooth selectors give the interior distributional statement. For selectors meeting the boundary, the closed-port balance pairing is 〈Bj,μa〉, agreeing with the diamond pairing on interior tests. The following auxiliary mixed formulation combines this primitive balance with a potential-based flux closure.

Let Ψ:Y→[0,+∞] be a proper, convex, lower-semicontinuous flux power functional with Ψ(0)=0, and let μ∈V be the energetic effort. Define

(59) $$L_{\mu }\left(v,j,\lambda \right):=\Psi \left(j\right)+{\langle v,\mu \rangle }_{V^{*},V}-{\langle v+Bj,\lambda \rangle }_{V^{*},V},\;\lambda \in V.$$

A triple (v,j,λ) is stationary when

(60a) $$\begin{array}{cccc} {\langle w,\mu -\lambda \rangle }_{V^{*},V} & =0\; & \; & \mathrm{for}\;\mathrm{every}\;w\in V^{*}, \end{array}$$

(60b) $$\begin{array}{cccc} {\langle v+Bj,\eta \rangle }_{V^{*},V} & =0\; & \; & \mathrm{for}\;\mathrm{every}\;\eta \in V, \end{array}$$

(60c) $$\begin{array}{cccc} \Psi \left(k\right)-\Psi \left(j\right)+{\left(k-j,G\lambda \right)}_{Y} & \geq 0\; & \; & \mathrm{for}\;\mathrm{every}\;k\in Y. \end{array}$$

**Theorem 6** (Mixed stationarity)**.** *Conditions *(60)* are equivalent to*

(61a) $$\begin{array}{cccc} \lambda & =\mu & \; & \mathrm{in}\;V, \end{array}$$

(61b) $$\begin{array}{cccc} v+Bj & =0 & \; & \mathrm{in}\;V^{*}, \end{array}$$

(61c) $$\begin{array}{cccc} -G\mu & \in \partial \Psi (j) & \; & \mathrm{in}\;Y. \end{array}$$

**Proof.**  The first two stationarity conditions give (61a) and (61b) because the corresponding test spaces separate points. The third condition is the subgradient inequality −Gλ∈∂Ψ(j); use (61a). Every step is reversible. □

The three variations play different roles. The rate direction identifies the multiplier with the energetic effort, the multiplier direction imposes conservation against the full test space *V*, and the flux inequality supplies the constitutive contact. Keeping these directions separate is what yields an unweighted balance in $V^{*}$.

Testing (61b) by 1∈V gives

(62) $${\langle v,1\rangle }_{V^{*},V}=0,$$

since G1=0. Thus, $v=\dot{c}$ conserves total mass.

## 6. Energy Balance and Process Power

Let

(63) $$c\in W^{1,1}\left(0,T;V^{*}\right),\;\mu \in L^{1}\left(0,T;V\right),$$

and let E be a chemical free energy such that E(c(·)) is absolutely continuous and

(64) $$\frac{\;d}{\;dt}E\left(c\left(t\right)\right)={\langle \dot{c}\left(t\right),\mu \left(t\right)\rangle }_{V^{*},V}\;\mathrm{for}\;\mathrm{almost}\;\mathrm{everywhere}\;t.$$

For nonconvex weak evolutions, this chain rule is a model-dependent hypothesis [8,34].

Let $\Psi_{t}:Y\to \left[0,+\infty \right]$ be a measurable family of proper, convex, lower-semicontinuous flux power functions with $\Psi_{t}\left(0\right)=0$, and set

(65) $$e\left(t\right):=-G\mu \left(t\right),\;D\left(t\right):=\Psi_{t}\left(j\left(t\right)\right)+\Psi_{t}^{*}\left(e\left(t\right)\right).$$

Assume that all terms below are integrable.

**Proposition 2** (Energy–power equivalence)**.** *Suppose*

(66) $$\dot{c}+Bj=0\;\mathrm{in}\;V^{*}\;\mathrm{for}\;\mathrm{almost}\;\mathrm{everywhere}\;t.$$

*Then, the constitutive contact*

(67) $$e\left(t\right)\in \partial \Psi_{t}\left(j\left(t\right)\right)$$

*is equivalent to*

(68) $$E\left(c\left(t\right)\right)+\int_{s}^{t}D\left(\tau \right)\;d\tau =E\left(c\left(s\right)\right)\;\left(0\leq s\leq t\leq T\right).$$

*Equivalently, under the same balance and chain-rule hypotheses, it suffices to assume *(68) *with equality replaced by ≤ for s=0 and t=T; the Fenchel gap then vanishes almost everywhere, and equality follows on every subinterval.*

**Proof.**  By (64), (66), and (56),

(69) $$\frac{\;d}{\;dt}E\left(c\left(t\right)\right)=-\langle Bj,\mu \rangle ={\left(j,G\mu \right)}_{Y}=-{\left(e,j\right)}_{Y}.$$

Fenchel–Young gives the nonnegative gap

(70) $$\Psi_{t}\left(j\right)+\Psi_{t}^{*}\left(e\right)-{\left(e,j\right)}_{Y}\geq 0,$$

with equality exactly under (67). Integration proves both directions. This is the standard energy–power argument for generalized gradient systems [7,8]. □

For an open normal port, assume j∈H(div;Ω), let $g=\gamma_{n}j$, and write the balance as $\dot{c}+\mathrm{div}j=0$. The Green identity is

(71) $$\int_{\Omega }\mu \mathrm{div}j\;dx+\int_{\Omega }j\cdot \nabla \mu \;dx={\langle g,\gamma \mu \rangle }_{H^{-1/2},H^{1/2}}.$$

Under the constitutive contact (67) and with integrable boundary power,

(72) $$E\left(c\left(t\right)\right)+\int_{s}^{t}D\left(\tau \right)\;d\tau +\int_{s}^{t}\langle g(\tau ),\gamma \mu (\tau )\rangle \;d\tau =E\left(c\left(s\right)\right).$$

With outward normal orientation, the last term is chemical power leaving the domain. The operator *B* in (55) is reserved for the closed-port realization.

Along a closed trajectory satisfying (67), Fenchel equality yields

(73) $$D\left(t\right)={\left(e\left(t\right),j\left(t\right)\right)}_{Y}=-\int_{\Omega }j\cdot \nabla \mu \;dx\geq 0.$$

For spatially constant selectors, combining (69) with the spending rule gives

(74) $$D\left(t\right)\delta t_{s}-D\left(t\right)\delta t_{c}=D\left(t\right)\delta t_{s}+{\langle \dot{c}\left(t\right)\delta t_{c},\mu \left(t\right)\rangle }_{V^{*},V}.$$

If the local power density

(75) p(x,t):=−j(x,t)·∇μ(x,t)

belongs to $C(I;L^{1}\left(\Omega \right))$, Theorem 2 gives

(76) $${\left.\frac{\;d}{\;d\varepsilon }\right|}_{\varepsilon =0}\Gamma_{p}\left[t+\varepsilon \delta t_{s},t+\varepsilon \delta t_{c}\right]=\int_{\Omega }p\left(x,t\right)\left(\delta t_{s}-\delta t_{c}\right)\;dx.$$

Equation (76) describes the local internal-conversion increment; the full diffusion port retains the two channel pairings in (38). The scalar closed-port identification above applies to spatially constant selectors.

## 7. A Synchronized Thermo-Diffusion Functional

This section realizes the preceding structures within one scalar functional on a product space. Storage variables, balance variables, and stored histories remain independent during differentiation and are synchronized only afterward. Stationarity is imposed along the declared direction space. In this auxiliary mixed realization, the multiplier supplies the full weak balance test space alongside the calibrated state–history channels. The multiplier and flux potentials are specific to this realization.

### 7.1. Storage, Mixed, and History Blocks

Let Ω be a bounded $C^{1}$ domain and use closed normal ports in the derivation. Introduce an energetic concentration $c_{E}$, a kinematic concentration $c_{K}$, an energetic temperature $\theta_{E}$, and a mixed-block temperature $\theta_{M}$. Define

(77) $$F\left(c_{E},\theta_{E}\right):=\int_{\Omega }\psi \left(c_{E},\nabla c_{E},\theta_{E}\right)\;dx.$$

For smooth fields, write

(78) $$D_{c}F\left(c_{E},\theta_{E}\right)\left[\eta \right]=\int_{\Omega }\mu_{F}\eta \;dx,\;\mu_{F}=\partial_{c}\psi -\mathrm{div}\partial_{\nabla c}\psi ,$$

with the corresponding natural boundary condition.

Let $\mu_{E}$ be the effort in the energetic–kinematic bridge, let $\mu_{M}$ be the effort in the mixed block, and let $\left(\bar{\mu },\bar{j},\bar{q}\right)$ denote fixed stored-history copies. Let *s* be the entropy density, *v* the concentration rate, and λ an independent balance multiplier. For nonnegative convex $C^{1}$ flux power densities $r_{d}\left(x,\cdot \right)$ and $r_{q}\left(x,\cdot \right)$ with $r_{d}\left(x,0\right)=r_{q}\left(x,0\right)=0$, use a fixed reference temperature $\theta_{*}>0$ to scale the thermal terms to diffusion-power units and define

(79) $$\begin{array}{cc} M_{\mu_{M},\theta_{M}}\left(v,j,q,\lambda \right):=\int_{\Omega }[ & r_{d}\left(x,j\right)+\frac{r_{q}\left(x,q\right)}{\theta_{*}}+\mu_{M}v\\ \; & -\lambda \left(v+\mathrm{div}j\right)+\frac{q\cdot \nabla \theta_{M}}{\theta_{*}}]\;dx. \end{array}$$

The bridge is

(80) $$B\left(c_{E},c_{K};\mu_{E}\right):=\int_{\Omega }\mu_{E}\left(c_{K}-c_{E}\right)\;dx.$$

It vanishes after synchronization but has nonzero derivatives in the two independent concentration directions.

For endpoint fields $t_{c},t_{s}$, define the stored histories

(81) $$H_{\bar{q}}\left[t_{s}\right]:=\int_{\Omega }\int_{t_{0}}^{t_{s}\left(x\right)}\mathrm{div}\bar{q}\left(x,\tau \right)\;d\tau \;dx$$

and

(82) $$\begin{array}{cc} H_{\bar{\mu }\bar{j}}\left[t_{c},t_{s}\right]:= & \int_{\Omega }\int_{t_{0}}^{t_{c}\left(x\right)}\bar{\mu }\mathrm{div}\bar{j}\;d\tau \;dx\\ \; & +\int_{\Omega }\int_{t_{0}}^{t_{s}\left(x\right)}\bar{j}\cdot \nabla \bar{\mu }\;d\tau \;dx. \end{array}$$

At synchronized endpoints and history copies, (82) reconstructs the accumulated history of div(μj).

For a fixed positive scaling parameter $\tau_{*}$ with units of time, set

(83) $$\begin{array}{cc} {\hat{\Pi }}_{t}:= & \;F\left(c_{E},\theta_{E}\right)+\int_{\Omega }\theta_{E}s\;dx+B\left(c_{E},c_{K};\mu_{E}\right)\\ \; & +\tau_{*}M_{\mu_{M},\theta_{M}}\left(v,j,q,\lambda \right)+H_{\bar{q}}\left[t_{s}\right]+H_{\bar{\mu }\bar{j}}\left[t_{c},t_{s}\right]. \end{array}$$

The physical synchronization set at time *t* is

(84) $$\begin{array}{c} c_{E}=c_{K}=c,\;\theta_{E}=\theta_{M}=\theta ,\;\mu_{E}=\mu_{M}=\bar{\mu }=\mu ,\\ \bar{j}=j,\;\bar{q}=q,\;v=\dot{c},\;t_{c}=t_{s}=t. \end{array}$$

Denote this set by $S_{t}$, and set $\Pi_{t}={\hat{\Pi }}_{t}{|}_{S_{t}}$ after differentiation.

### 7.2. Admissible Directions and First Variation

At a point of $S_{t}$, take

(85) $$h=(\eta ,\vartheta ,w,k_{d},k_{q},\ell ,\delta t_{c},\delta t_{s}),$$

where the scalar directions belong to $C_{c}^{\infty }\left(\Omega \right)$ and the flux directions to $C_{c}^{\infty }\left(\Omega ;R^{d}\right)$ in the bulk calculation. The nonzero field components are

(86) $$\begin{array}{c} \delta c_{E}=\eta ,\;\delta c_{K}=v\;\delta t_{c},\;\delta \theta_{E}=\vartheta ,\;\delta s=\dot{s}\;\delta t_{s},\\ \delta v=w,\;\delta j=k_{d},\;\delta q=k_{q},\;\delta \lambda =\ell . \end{array}$$

The endpoint components of h are $\delta t_{c}$ and $\delta t_{s}$. The copies $\mu_{E},\mu_{M},\theta_{M}$ and the stored histories $\left(\bar{\mu },\bar{j},\bar{q}\right)$ remain fixed in this direction. Define

(87) $$\delta \Pi_{t}\left[h\right]:=D{\hat{\Pi }}_{t}{|}_{S_{t}}\left[h\right].$$

The derivative is evaluated in the ambient product space before restriction to $S_{t}$; the admissible directions need not be tangent to $S_{t}$.

The vertical state variation η and the trajectory-generated direction $v\;\delta t_{c}$ are independent before synchronization. The energetic and kinematic copies separate these operations. The spatial variation and integration by parts in (78) precede evaluation in the channel direction.

A sufficient classical setting is $\psi \in C^{2}$, $c,\theta ,s\in C^{1}\left(I;C^{2}\left(\bar{\Omega }\right)\right)$ with θ>0, and $\mu ,j,q\in C(I;C^{1}\left(\bar{\Omega }\right))$, likewise for the corresponding copies. The stored densities $\mathrm{div}\bar{q}$, $\bar{\mu }\mathrm{div}\bar{j}$, and $\bar{j}\cdot \nabla \bar{\mu }$ then belong to $C(I;L^{1})$, as required by the scalar terminal rules. Closed normal ports and the natural microstress condition $\partial_{\nabla c}\psi \cdot n=0$ remove the boundary terms. The finite-energy interpretation is stated after (98).

**Theorem 7** (First variation of the synchronized functional)**.** *Assume that ψ is continuously differentiable in its displayed variables, that $r_{d}$ and $r_{q}$ are continuously differentiable in their flux variables, and that the synchronized fields, histories, and directions have the regularity required by the integrations and terminal derivatives above. In the closed-port calculation, the normal components of the flux variations vanish. Then,*

(88) $$\begin{array}{cc} \delta \Pi_{t}\left[h\right]= & \int_{\Omega }[\left(\mu_{F}-\mu \right)\eta +\left(\partial_{\theta }\psi +s\right)\vartheta +\mu \left(v+\mathrm{div}j\right)\delta t_{c}\\ \; & \;+\left(\theta \dot{s}+\mathrm{div}q+j\cdot \nabla \mu \right)\delta t_{s}]\;dx\\ \; & +\tau_{*}\int_{\Omega }[\left(\mu -\lambda \right)w+\left(\partial_{j}r_{d}+\nabla \lambda \right)\cdot k_{d}\\ \; & \;+\theta_{*}^{-1}\left(\partial_{q}r_{q}+\nabla \theta \right)\cdot k_{q}-\left(v+\mathrm{div}j\right)\ell ]\;dx. \end{array}$$

**Proof.**  The storage and bridge blocks contribute

$$\int_{\Omega }\left[\mu_{F}\eta +\left(\partial_{\theta }\psi +s\right)\vartheta +\theta \dot{s}\;\delta t_{s}+\mu \left(v\delta t_{c}-\eta \right)\right]\;dx.$$

The mixed block gives the second integral in (88); the closed-port integration by parts changes $-\int_{\Omega }\lambda \mathrm{div}k_{d}\;dx$ into $\int_{\Omega }\nabla \lambda \cdot k_{d}\;dx$. The terminal derivatives of (81) and (82) are

$$\int_{\Omega }\mathrm{div}q\;\delta t_{s}\;dx,\;\int_{\Omega }\left(\mu \mathrm{div}j\;\delta t_{c}+j\cdot \nabla \mu \;\delta t_{s}\right)\;dx,$$

by Theorem 4. Adding the contributions proves the formula. □

The storage and bridge terms generate conjugacy and the trajectory-based species power, the mixed block provides the independently tested balance and flux laws, and the locked histories supply the two terminal channels.

**Theorem 8** (Generated thermo-diffusion structure)**.** *Under the hypotheses of Theorem 7, the directional stationarity condition*

(89) $$\delta \Pi_{t}\left[h\right]=0\;\mathrm{for}\;\mathrm{every}\;\mathrm{admissible}\;h$$

*is equivalent to*

(90a) $$\begin{array}{cccc} \mu & =\frac{\delta F}{\delta c}=\partial_{c}\psi -\mathrm{div}\partial_{\nabla c}\psi ,\; & \; & \mathrm{energetic}\;\mathrm{conjugacy}, \end{array}$$

(90b) $$\begin{array}{cccc} s & =-\partial_{\theta }\psi ,\; & \; & \mathrm{thermal}\;\mathrm{conjugacy}, \end{array}$$

(90c) $$\begin{array}{cccc} v+\mathrm{div}j & =0,\; & \; & \mathrm{species}\;\mathrm{balance}, \end{array}$$

(90d) $$\begin{array}{cccc} \lambda & =\mu ,\; & \; & \mathrm{balance}\;\mathrm{effort}, \end{array}$$

(90e) $$\begin{array}{cccc} \partial_{j}r_{d}\left(x,j\right)+\nabla \mu & =0,\; & \; & \mathrm{diffusion}\;\mathrm{law}, \end{array}$$

(90f) $$\begin{array}{cccc} \partial_{q}r_{q}\left(x,q\right)+\nabla \theta & =0,\; & \; & \mathrm{heat}‐\mathrm{flux}\;\mathrm{law}, \end{array}$$

(90g) $$\begin{array}{cccc} \theta \dot{s}+\mathrm{div}q+j\cdot \nabla \mu & =0,\; & \; & \mathrm{entropy}\;\mathrm{channel}. \end{array}$$

*The coefficient μ(v+divj) then vanishes by the independently generated balance; it is not divided by μ.*

**Proof.**  Independence of $\eta ,\vartheta ,w,k_{d},k_{q},\ell$, and $\delta t_{s}$ in (88) gives (90). The $\delta t_{c}$ coefficient is the weighted channel and follows from (90c). Conversely, substitution of (90) into (88) makes every coefficient vanish. □

Under the hypotheses of Theorem 5, the weighted selector coefficient μ(v+divj) in (88) already implies the local species balance for regular fields. The independent multiplier direction *ℓ* is retained because it yields the same balance in $V^{*}$ without the strong regularity and isolated-zero-state assumptions.

For scalar endpoints in a closed port, (27) gives the exact history identity

(91) $$H_{\mu j}\left[t_{c},t_{s}\right]=H_{D_{d}}\left(t_{c}\right)-H_{D_{d}}\left(t_{s}\right)=-\Gamma_{D_{d}}\left(t_{s},t_{c}\right).$$

The split-port history vanishes at synchronized endpoints, whereas the accumulated power $H_{D_{d}}\left(t\right)$ is generally nonzero. Identity (91) relates their calibrated channel increments with opposite orientations.

The two directly reusable terminal formulas are present in the same functional:

(92) $$\delta^{\mathrm{ch}}\int_{t_{0}}^{t}\mathrm{div}\left(\mu j\right)\;d\tau =\mu \mathrm{div}j\;\delta t_{c}+j\cdot \nabla \mu \;\delta t_{s},$$

and, for a closed diffusion port and spatially constant selectors, with $D_{d}$ from (42),

(93) $$\delta^{\mathrm{ch}}\int_{t_{0}}^{t}D_{d}\left(\tau \right)\;d\tau =D_{d}\left(t\right)\delta t_{s}-D_{d}\left(t\right)\delta t_{c}.$$

In the terminal energy convention of (83), replacing the closed split port by receiver-positive spending requires the opposite sign, $-\delta^{\mathrm{ch}}H_{D_{d}}$. These are alternative representations of the same process power.

For the quadratic flux power densities

(94) $$r_{d}\left(x,j\right)=\frac{1}{2}j\cdot M^{-1}j,\;r_{q}\left(x,q\right)=\frac{1}{2}q\cdot K^{-1}q,$$

where M and K are symmetric positive definite, (90e)–(90f) give

(95) j=−M∇μ,q=−K∇θ.

Hence,

(96) $$\theta \dot{s}+\mathrm{div}q=\nabla \mu \cdot M\nabla \mu \geq 0.$$

For smooth θ>0, the entropy-flux form is

$$\dot{s}+\mathrm{div}\left(q/\theta \right)=\frac{-j\cdot \nabla \mu }{\theta }+q\cdot \nabla \left(1/\theta \right)\geq 0.$$

The term divq represents heat transport; the two production terms follow from the same diffusion and Fourier closures.

At finite energy, the conserved-variable channel in (88) is replaced by

(97) $${\langle v,\mu \delta t_{c}\rangle }_{V^{*},V}+\langle \mu ⋄\mathrm{div}j,\delta t_{c}\rangle ,$$

and the entropy-side diffusion term remains

(98) $$\int_{\Omega }j\cdot \nabla \mu \;\delta t_{s}\;dx.$$

Take $v,\dot{s}\in V^{*}$, μ,θ∈V, j,q∈Y, and selectors $a=\delta t_{c}$, $b=\delta t_{s}\in W^{1,\infty }\left(\Omega \right)$. Using the closed-port extension defined in Section 5, (97) equals 〈v+Bj,μa〉. The full entropy channel is

(99) $$\langle \dot{s},\theta b\rangle +\langle Bq,b\rangle +\int_{\Omega }j\cdot \nabla \mu \;b\;dx.$$

The product estimate (58) makes these pairings meaningful. For histories, assume the fixed-test pairings are locally integrable in time, as in Section 4; their terminal derivatives then hold almost everywhere. This gives the tested counterpart of the classical first variation. The weighted species channel is the energetic projection of the primitive weak balance.

## 8. Cahn–Hilliard Realization

This section treats the isothermal chemical subsystem.

Let d≤3, let κ>0, and let $F\in C^{1}\left(R\right)$ be bounded below. Assume

(100) $$|F^{\prime }\left(r\right)|\leq C_{F}{\left(1+|r\right|}^{q}),\;1\leq q\leq 5,$$

so that the weak nonlinear term is defined for $H^{1}\left(\Omega \right)$ fields in three dimensions. Define

(101) $$E_{\mathrm{CH}}\left(c\right):=\int_{\Omega }\left(F\left(c\right)+\frac{\kappa }{2}{\left|\nabla c\right|}^{2}\right)\;dx.$$

A weak chemical potential $\mu \in H^{1}\left(\Omega \right)$ satisfies

(102) $$\int_{\Omega }\mu \zeta \;dx=\int_{\Omega }F^{\prime }\left(c\right)\zeta \;dx+\kappa \int_{\Omega }\nabla c\cdot \nabla \zeta \;dx\;\mathrm{for}\;\mathrm{every}\;\zeta \in H^{1}\left(\Omega \right).$$

For smooth fields, this is $\mu =F^{\prime }\left(c\right)-\kappa \Delta c$ with $\partial_{n}c=0$.

Assume

(103) $$c\in W^{1,1}\left(0,T;H^{1}{\left(\Omega \right)}^{*}\right)\cap L^{\infty }\left(0,T;H^{1}\left(\Omega \right)\right),\;\mu \in L^{2}\left(0,T;H^{1}\left(\Omega \right)\right),$$

and absolute continuity of $E_{\mathrm{CH}}\left(c\left(\cdot \right)\right)$, together with the chain rule

(104) $$\frac{\;d}{\;dt}E_{\mathrm{CH}}\left(c\left(t\right)\right)={\langle \dot{c}\left(t\right),\mu \left(t\right)\rangle }_{H^{1}{\left(\Omega \right)}^{*},H^{1}\left(\Omega \right)}\;\mathrm{for}\;\mathrm{almost}\;\mathrm{everwhere}\;t.$$

These hypotheses isolate the energy regularity required below; classical weak-solution results provide concrete sufficient assumptions, while energetic formulations with dynamic boundary effects illustrate the additional trace terms required for open interfaces [22,35,36].

Let $M\in L^{\infty }\left(\Omega ;R^{d\times d}\right)$ be symmetric and uniformly elliptic:

(105) $$m_{0}{\left|\xi \right|}^{2}\leq \xi \cdot M\left(x\right)\xi \leq m_{1}{\left|\xi \right|}^{2}$$

for some $0<m_{0}\leq m_{1}$ and almost every *x*. Set

(106) $$\Psi_{\mathrm{CH}}\left(j\right):=\frac{1}{2}\int_{\Omega }j\cdot M^{-1}j\;dx$$

and

(107) $$L_{\mathrm{CH}}\left(v,j,\lambda \right):=\Psi_{\mathrm{CH}}\left(j\right)+\langle v,\mu \rangle -\langle v+Bj,\lambda \rangle .$$

**Theorem 9** (Cahn–Hilliard realization)**.** For a rate $v\in H^{1}{\left(\Omega \right)}^{*}$, the following are equivalent:

(i) *There exist $j\in L^{2}\left(\Omega ;R^{d}\right)$ and $\lambda \in H^{1}\left(\Omega \right)$ stationary for *(107)*;*
(ii)
(108a) $$\begin{array}{cccc} v+Bj & =0 & \; & \mathrm{in}\;H^{1}{\left(\Omega \right)}^{*}, \end{array}$$
(108b) $$\begin{array}{cccc} j & =-M\nabla \mu & \; & \mathrm{in}\;L^{2}\left(\Omega ;R^{d}\right), \end{array}$$
(108c) $$\begin{array}{cccc} \lambda & =\mu & \; & \mathrm{in}\;H^{1}\left(\Omega \right); \end{array}$$
(iii)
(109) $$\langle v,\eta \rangle +\int_{\Omega }M\nabla \mu \cdot \nabla \eta \;dx=0\;\mathrm{for}\;\mathrm{every}\;\eta \in H^{1}\left(\Omega \right).$$

*For smooth fields and $v=\dot{c}$, this gives*

(110) $$\dot{c}=\mathrm{div}\left(M\nabla \mu \right),\;\mu =F^{\prime }\left(c\right)-\kappa \Delta c,$$

*with M∇μ·n=0 and $\partial_{n}c=0$.*

**Proof.**  The equivalence of (i) and (ii) follows from Theorem 6. Substitution of (108b) into (108a) gives (109); the converse is obtained by defining j through (108b) and setting λ=μ. The strong form follows whenever the displayed derivatives and traces exist. □

**Corollary 2** (Mass, energy, and terminal channels)**.** *Let $v=\dot{c}$, and assume (103)–(104) and the equivalent conditions of Theorem 9. Then,*

(111) $$\langle \dot{c}\left(t\right),1\rangle =0\;\mathrm{for}\;\mathrm{almost}\;\mathrm{everywhere}\;t,$$

*and*

(112) $$E_{\mathrm{CH}}\left(c\left(t\right)\right)+\int_{\Omega }\int_{s}^{t}\nabla \mu \cdot M\nabla \mu \;d\tau \;dx=E_{\mathrm{CH}}\left(c\left(s\right)\right).$$

*At almost every time,*

(113) $$\mathrm{div}\left(\mu j\right)=\mu ⋄\mathrm{div}j+j\cdot \nabla \mu \;\mathrm{in}\;D^{\prime }\left(\Omega \right),$$

*with*

(114) −j·∇μ=∇μ·M∇μ≥0.

*Consequently, the local diffusion-port rule is (38), and the closed scalar spending rule is*

(115) $$\delta^{\mathrm{ch}}\int_{t_{0}}^{t}D_{\mathrm{CH}}\left(\tau \right)\;d\tau =D_{\mathrm{CH}}\left(t\right)\left(\delta t_{s}-\delta t_{c}\right),\;D_{\mathrm{CH}}\left(t\right):=\int_{\Omega }\nabla \mu \cdot M\nabla \mu \;dx.$$

**Proof.**  Test (109) by 1 to obtain (111) and by μ to obtain the differential form of (112). Equations (113)–(114) follow from Definition 3 and (108b). The terminal statement is Theorem 1 applied to $D_{\mathrm{CH}}$. □

For the quartic double well

(116) $$F\left(c\right)=\frac{1}{4}{\left(c^{2}-1\right)}^{2},$$

relation (102) becomes

(117) $$\int_{\Omega }\mu \zeta \;dx=\int_{\Omega }c\left(c^{2}-1\right)\zeta \;dx+\kappa \int_{\Omega }\nabla c\cdot \nabla \zeta \;dx.$$

For constant isotropic mobility, elimination of μ gives the classical fourth-order Cahn–Hilliard equation [18,20].

## 9. Discussion and Conclusions

The analysis identifies the mathematical content of the two channel rules used in path-dependent entropic Lagrangians. Endpoint calibration and cocycle additivity determine the oriented spending differential, while local selector fields provide the test-function localization required for field equations. The diffusion port has an H(div) form with normal-trace boundary power and a finite-energy form based on μ⋄divj.

For regular diffusion models, the weighted species channel yields local conservation when every persistent zero-potential state is dynamically isolated in the admissible state class. Local energies with discrete critical states satisfy this condition directly. For gradient energies, the local tangent-space check is essential. The auxiliary mixed formulation supplies the primitive weak balance without these strong recovery assumptions.

The synchronized mixed thermo-diffusion functional places storage conjugacy, local conservation, flux laws, entropy production, and terminal routing in one directional stationarity structure. The Cahn–Hilliard specialization verifies the chemical balance, energy identity, and weak terminal channels at finite-energy regularity. Subsequent applications can invoke the spending rule, the diffusion-port rule, and the constitutive recovery theorem after specifying the selector class, boundary port, and the structure of zero-potential states.

## Data Availability

No new data were created or analyzed in this study. Data sharing is not applicable to this article.

## Appendix A. Auxiliary Multichannel Extension

Let $D_{e}\in L^{1}\left(t_{0},T\right)$ have primitives $H_{e}$, and let $A\in R^{m\times q}$ satisfy

(A1) $$1^{T}A=0.$$

Define

(A2) $$\Gamma_{A}\left(t_{1},\ldots ,t_{m}\right):=\sum_{e=1}^{q}\sum_{\alpha =1}^{m}A_{\alpha e}H_{e}\left(t_{\alpha }\right).$$

At a common Lebesgue point *t*,

(A3) $$D\Gamma_{A}\left(t1\right)\left[\xi \right]=\left(AD\left(t\right)\right)\cdot \xi ,\;D\Gamma_{A}\left(t1\right)\left[\xi 1\right]=0.$$

Thus, each column assigns one internal-conversion process power to a zero-sum family of terminal channels. This is the direct multichannel extension of (13); graph incidence matrices provide a standard realization [9].

## Appendix B. Bounded-Variation Extension

For $\mu \in BV\left(\Omega \right)\cap L^{\infty }\left(\Omega \right)$ and $j\in DM^{\infty }\left(\Omega \right)$, the Anzellotti pairing (j,Dμ) is the Radon measure defined distributionally by

(A4) $$\langle (j,D\mu ),\varphi \rangle :=-\int_{\Omega }\mu^{*}\varphi \;d\left(\mathrm{div}j\right)-\int_{\Omega }\mu j\cdot \nabla \varphi \;dx,\;\varphi \in C_{c}^{1}\left(\Omega \right).$$

Here, $DM^{\infty }\left(\Omega \right)$ consists of essentially bounded vector fields whose divergence is a finite Radon measure, and $\mu^{*}$ is the precise representative, equal to the mean of the two approximate traces on the jump set. It satisfies

(A5) $$\mathrm{div}\left(\mu j\right)=\mu^{*}\mathrm{div}j+\left(j,D\mu \right),\;|\left(j,D\mu \right)|\leq {∥j∥}_{L^{\infty }}\left|D\mu \right|.$$

The absolutely continuous part is $j\cdot \nabla \mu \;L^{d}$, while the singular part retains interfacial power on jump and Cantor sets [24,25,26,37]. This extension is auxiliary to the $H^{1}\times L^{2}$ theory used in the main text; the underlying BV notation and fine properties are as in [38].
